# Nutritional management of hyperlipaemia in a jenny: a brief report

**DOI:** 10.1007/s11259-024-10460-7

**Published:** 2024-07-04

**Authors:** S. Morrone, S. Sechi, C. Carta, A. Senes, R. Cocco, M. L. Pinna Parpaglia, E. Sanna Passino, M. G. Cappai

**Affiliations:** Veterinary Teaching Hospital of the Department of Veterinary Medicine of the University of Sassari, via Vienna no. 2, Sassari, 07100 Italy

**Keywords:** BCS, Emaciation, Nutritional plan, Refeeding, Donkey

## Abstract

An adult jenny (5-years-old, non-pregnant) was presented to the Veterinary Teaching Hospital (VTH) of the University of Sassari, with a recent history of appetite loss, extreme underweight condition and reluctance to move. On physical inspection, emaciation [body condition score, BCS: 3/9], muscular waste [muscular condition score, MCS: 1/5], loose/running faeces [faecal score, FS: 2/8], and a general state of mild dehydration were found. Blood analyses outlined a general undernourishment condition [circulating albumins, ALB: 17.6 g/L (21.6–31.6 g/L)] with underlying systemic inflammatory profile and moderate increase in circulating enzymes to explore liver function [aspartate amino-transferase, AST: 657 u/L (279–430 u/L); alanine amino-transferase ALT: 60 u/L (5–14 u/L); gamma-glutamyl-transferase, γ-GT: 87 IU/L (14–69 IU/L); total bilirubin close to the upper limit, TB: 0.20 mg/dL(0.07–0.21 mg/dL)]and hyperlipaemia [TG: 8.70 mmol/L (0.60–2.87 mmol/L)], following fat depots mobilisation, with total cholesterol closed to the lower limit of the physiological range. Hyper-phosphataemia was linked to haemolytic anaemia [P:1.81 mmol/L (0.77–1.39 mmol/L) and red blood cells, RBC: 4.14 10^12^/L (4.40–7.10 10^12^)] aligned with the TB to the upper limit. On ultrasound abdominal imaging, enlarged and hyper-echogenic liver was observed. Based on the clinical evaluation, a condition of hepatic lipidosis was diagnosed, requiring dedicated nutritional treatment to solve the extreme emaciation along with the metabolic disorder in support of medical therapy. A two-step feeding protocol was planned to support treatments aiming at immediate re-hydration (Ringer lactate solution 2 ml/kg/8 h). The nutritional objectives were meant at first to restart the voluntary feed intake. Gradual increasing energy provision through a palatable hay-based diet was planned to cover one fourth of daily metabolizable energy requirement calculated on the expected metabolic weight, adjusted according to the daily intake of feed and clinical condition. At the conclusion of this first 7-day phase, circulating blood parameters were closer to the reference values and the BCS moved from 3 to 4 out of 9. Bowel motility was restored, and faecal score improved (4/8). In the second phase, allowance to pasture and a combination diet with compound mixed feed were designed. Within four weeks of starting the nutritional plan, blood parameters were re-established to reference values. The gradual feed provision calculated in this two-phase approach proved successful in support of the overall clinical improvement observed after four weeks of treatment, in a severely undernourished jenny with compromised liver functions.

## Background

The donkey is a hindgut fermenter that has adapted to have a constant provision of fibrous vegetal material with a transit time dependent on fibre fraction proportions and particle size of the original diet, through the different gastro-intestinal tracts (GITs) (Burden and Thiemann 2015). Donkeys are highly efficient in digesting fibrous feedstuffs which may turn to be of low nutritional value to horses, and their energy requirements are comparatively lower than those of a horse in proportion to the size, with levels ranging from 50 to 75% of the horse’s energy demands at maintenance(Smith and Burden 2013). However, recent nutritional approaches and interest in donkey applied nutrition are leading to acknowledge species-specific requirements in consideration of the diverse utilisation of feeding material, undergoing fermentative processes by microbial populations. Breed specific acquaintances on the metabolic profile at different physiological stages were also considered for nutritional state estimation (Cappai et al. 2013, 2017, 2021). Health issues of nutritional origin in donkeys include obesity, metabolic or hormonal anomalies, hyperlipaemia, and laminitis, often caused by wrong feed supply(Burden et al. 2011). On the other hand, dietary errors have been associated with a variety of disorders, including those affecting teeth, stomach, liver, small and large intestine, along with the involvement of different organs simultaneously, other than those involved in the digestive functions(Burden and Bell 2019). Donkeys are particularly susceptible to developing hyperlipaemia, where sustained negative energy balance (NEB) from unmet requirements leads to body fat mobilization, which on a long-term low energy intake may cause the depletion of animal energy storage, challenging liver function. Such metabolic pathway of energy partitioning from peripheral fat depots brought to the liver in charge of rearranging energy substrates to meet systemic needs, requires particular attention by the clinical nutritionist. In the animal with adequate daily energy intakes from the diet, energy and nutrients requirements are supplied. In the post-absorption phase, the endocrine regulation of glycemia through insulin favours glucose uptake from the bloodstream into the cells, where this is used. When energy intake with the diet is insufficient to meet requirements and prolonged, then downregulation of insulin production in front of low circulating glucose, triggers the lipase activation and fat mobilization from body depots. An increase in circulating free fatty acids in the bloodstream (milky serum), better referred to hyperlipidaemia, in particular, shows high plasma total triglyceride (TG, > 4.4 mmol/L). When fatty acids are mobilized from adipocytes (as chief tissue of energy storage), they are bound to serum albumin and transported in the bloodstream to the liver. As a result of liver overload, inefficient to convert fats into available energy substrate in the hepatocyte, the depletion of oxaloacetic acid in mitochondria and the incomplete beta-oxidation of fatty acids can lead to the presence of acetil-CoA molecules and the synthesis of acetoacetic acid, acetone and beta-hydroxybutirric acid (ketone bodies, as signalling mediators inducing loss of appetite; Masi et al. 2022). The excess of circulating fats getting to the liver can challenge the biochemical patterns leading to energy production, and when the hepatocyte is overloaded, this may lead hepatic lipidosis. The onset of hepatic lipidosis and severity of clinical conditions are triggered by the body condition and fatness of the animal facing the NEB. Obesity in the donkey is an issue and the mobilization of energy from fat depots can lead to excess of free fatty acids entering the liver. Such a metabolic condition has significant influence on donkey health status, for being potentially fatal, with a death rate reaching approximately 49%(Burden et al. 2011).

In view of the clinical significance of the metabolic status, nutritional assessment should be routinely performed as part of the clinical evaluation of the donkey(Cappai et al. 2013).

## Case presentation

A Sardinian jenny [5-years-old, body weight at the moment of presentation of 88 kg (breed normal weight ranging between100-130 kg, Pinna et al., 1990); BCS = 3/9; bilateral lameness, score 4] was referred to the Veterinary Teaching Hospital (VTH) of the Department of Veterinary Medicine, University of Sassari. The patient came from the Sanctuary of Sardo breed donkey, in the municipality of Ortueri, Sardinia (Italy). On physical examination, the animal showed jaundice, appetite loss, heart rate 60 beats per minute, respiratory rate 18 breath per minute, dehydration, reduced intestinal motility, remitting fever (> 39 °C), poor coat quality, and pale mucous membranes of natural openings, with slow capillary refill time (exceeding two seconds).The patient presented two wounds with a scarring process of second intention ongoing. Injuries were found on both sides of the limb, involving both the cutis and subcutis, with necrotic scar edges. Lesions needed curettage for purulent exudate. The assessment of nutritional status followed the method of Cappai and co-workers (2013): the body condition (BCS, 1 to 9-point scale; Henneke et al. 1983 ), indicated severe malnutrition. Hoof overgrowth was found, and limping scored [score of 4, following Hewetson et al. (2006) scale]. Faeces were loose with yellow/green colour, and harsh odour. No dental malocclusion or oral lesions causing pain that would have prevented sufficient feed intake and/or appropriate chewing activity were found.

The complete blood cell count (CBC) is reported in Table 1. Neutrophilia, lymphopenia, basophilia, low red blood cells (RBC) with elevated mean corpuscular haemoglobin concentration (MCHC) and thrombocytopenia were pointed out. An increase in triglycerides concentration (TG), aspartate transaminase (AST), and alanine transaminase (ALT) were also observed. The rapid weight loss involving lean mass catabolism may explain its elevated creatine phosphokinase (CPK) levels (Table 2). A condition of inadequate nutrition can be attributed for the decrease in the Ca/P ratio as well as the decline in albumin (ALB) levels. Based on CBC count and the presence of ticks, tick-borne infection was investigated. The jenny tested positive to *Rickettsia rickettsiae* at indirect immunofluorescence assay (positive at 1:128).

**Table 1:** Initial (D1), and subsequent CBC counts (D2, 1 month; D3, 6 months). Bold numbers indicate values dropping out of reference values

Item^a^	Reference values^b^	D1	Sampling time^c^ D2	D3
%NEUT (%)	23.0–59.0	**81.0**	58.7	48.3
%LYMPH (%)	34.0–69.0	**8.80**	30.2	39.6
%MONO (%)	0.00–7.50	5.00	3.50	3,60
%EOS (%)	0.94–9.10	1.90	5.60	**10.2**
%BASO (%)	0.00–0.50	**2.00**	**1.20**	0.10
#NEUT (10^9^/L)	2.40–6.30	**7.70**	5.81	4.99
#LYMPH (10^9^/L)	2.20–9.60	**0.84**	2.99	3.86
#MONO (10^9^/L)	0.00–0.75	0.48	0.35	0.36
#EOS (10^9^/L)	0.10–0.90	0.18	0.56	**0.92**
#BASO (10^9^/L)	0.00–0.07	**0.19**	0.12	0.04
WBC (10^9^/L)	6.20–15.0	9.51	9.91	9.82
RBC (10^12^/L)	4.40–7.10	**4.14**	3.70	4.76
HGB (g/L)	89.0–147	111	97.0	93.0
HCT (%)	24.3– 39.6	27.6	24.8	26.4
MCV (fl.)	53.0– 67.0	66.6	67.0	66.9
MCH (pg)	17.6–23.1	26.9	26.1	19.5
MCHC (g/L)	310–370	**404**	389	352
PLT (10^9^/L)	95.0–384	**82.0**	315	324

^a^Item: *WBC* White blood cells, *NEUT* Neutrophils, *LYMPH* Lymphocytes, *MONO* Monocytes, *EOS* Eosinophils, *BASO*  Basophils, *NEUT* Neutrophils, *RBC* Red Blood Cells, *HGB* Haemoglobin, *HCT* Haematocrit, *MCV* Mean corpuscular volume, *MCH* Mean cell haemoglobin, *MCHC* Mean corpuscular haemoglobin concentration, *PLT* Platelet. ^b^Reference values: (Burden et al. 2016). The bold value in the table indicates outside reference value. ^c^different sampling times, at start and at the follow up (D2, D3)

**Table 2:** Initial (D1), and subsequent biochemical analyses (D2, 1 month; D3, 6 months). Bold numbers indicate values dropping out of reference values

Item^a^	Reference values^b^	D1^c^	D2^c^	D3^c^
ALB (g/l)	21.5–31.6	**17.6**	**20.3**	26.3
ALP (IU/l)	98.0–252	112	133	140
TB (mg/dl)	0.07–0.21	0.20	0.20	0.20
Ca (mmol/l)	2.20–3.40	**0.50**	2.40	2.60
CREA (µmol/l)	53.0–118	**44.0**	77.0	81.0
P (mmol/l)	0.77–1.39	**1.81**	**1.74**	1.22
TP (g/l)	58.0–76.0	60.0	74.0	73.0
γ-GT (IU/l)	14.0–69.0	**87.0**	37.0	39.0
AST (u/l)	279–430	**657**	324	341
ALT (u/l)	5.00–14.0	**60.0**	**22.0**	12.0
UREA (mg/dl)	11.0–40.0	19.0	40.0	33.0
TC (mmol/l)	1.40–2.90	1.90	1.90	1.90
CPK (IU/L)	128–525	239	273	221
GLU (mmol/L)	3.89–5.28	5.11	**5.83**	5.08
TG (mmol/l)	0.60–2.80	**8.70**	0.60	0.90

^a^Item: *ALB* Albumin, *ALP* Alkaline phosphatase, *TB* Total Bilirubin, *CREA* Creatinine, *TP* Total proteins, *γ-GT* Gamma-glutamyl-transferase, *AST* Aspartate amino-transferase, *ALT* Alanine amino-transferase, *TC* Total cholesterol, *CPK* Creatine phosphokinase, *GLU* Glucose, *TG* Total triglycerides. ^b^ Reference values: (Caldin et al. 2005; Burden et al. 2016); The bold value in the table indicates outside reference value. ^c^ different sampling times, at start (D1) and at the follow up (D2, D3)

The liver was screened on abdominal ultrasound imaging. Increased organ volume and hyperechogenic parenchyma were pointed out. When hospitalised, the patient was treated with intravenous (IV) fluid therapy to re-establish appropriate hydration. Rehydration was carried out through venous access using a balanced electrolyte solution (Ringer lactate solution 2 ml/kg/8 h), considering a state of dehydration of 10%, based on the clinical examination. The wounds were treated three times per day with an ointment containing collagenase and chloramphenicol, followed by bandaging to prevent contamination. Anti-inflammatory, antipyretic, and analgesic medications were prompted with the administration of flunixin meglumine at a dosage of 1.1 mg/kg BWIM per day for five days. The route of administration, the dose and the methods of administration were carefully considered given the very serious clinical conditions of the subject. Flunixin dosage was meant to alleviate pain and limit inflammatory state, where recommendations were adjusted to the clinical condition and animal response, heavily challenged, above all as to hepatic function. As a preventative approach, omeprazole was also administered at a minimum dose of 1 mg/ Kg BW *per os*. Additionally, hoof trimming and anti-parasite therapy (Equalan Duo: 100 mg Praziquantel + 20 mg ivermectin) were carried out after parasitological examination with the modified McMaster method, using a sodium chloride (NaCl) supersaturated solution (specific gravity = 1.2) for flotation and an egg detection limit of 15 eggs per gram (EPG) of faeces. The results showed positivity for *Anoplochephala spp*. and a gastrointestinal strongyles count of 1005 EPG.

## Nutritional approach

The nutritional treatment was split into two different consequential phases. During the phase, the reduced appetite and severe malnutrition were corrected with planning the feed offer to stimulate as much as possible the voluntary intake of feed. Small increasing amounts of compound mixed feeds were offered at the hand and then hidden in the bottom of the feeder, beneath the hay. High quality, highly palatable hay (Table 3) and straw were offered. Hay was weighed and fed at an amount of 1.80 kg per day, to meet the jenny requirement to reach a BW of 110 kg(Martin-Rosset 2018). Straw was provided *ad libitum* as well as used bedding material in the box. Free access to pasture was assured in a paddock of 40 × 35 m. Additionally, the following vitamin supplements were administered parenterally daily during the first phase: 6 mg of cyanocobalamin, 500 mg of thiamine hydrochloride, 1 g of dl-acetylmethionine, 250 mg of l-carnitine, and 150 mg of dl-[7:a]-tocopherol. To improve animal movement and voluntary grazing activity, hoof overgrowth was corrected. The lame jenny had likely been fasting for a considerable period that produced a prolonged negative energy balance from insufficient feed intake. The animal consumed the hay to get the limited quantity of available concentrate. Starting with feed offer of 50 g/day mixed compound feed, this limit was reduced daily until it was completely substituted by a whole hay day. Animal was under constant monitoring as to daily intake. After the first week of hospitalization, the animal was able to consume hay and straw autonomously. The BCS increased from 3 to 4/9, and the weight recorded was 97 kg. Also, faecal score improved from an initial value of 2 out of 8 to a value of 4 out of 8.

Considering these observations, the second phase of nutritional treatment started. In this second stage, the purpose was to re-establish normal nutritional state. Hay was administered at an amount of 1.50 kg/d and straw was used as litter material. Moreover, the jenny continued to have unrestricted access to a spontaneous pasture in a small paddock. The nutritional components of the mixed compound feed are detailed in Table 3 and were based on the estimation of energy needs (Martin-Rosset 2018) and assuming an optimum weight of 110 kg (Yılmaz et al. 2012):

$$\begin{aligned}\mathrm{DEm}\:&=\:109.94\;\mathrm{Kcal}\ast110^{0.75}=3734.22\;\mathrm{Kcal}\:\\&=\:3.73\;\mathrm{Mcal}\;\mathrm{required}\;\mathrm{per}\;\mathrm{day}\end{aligned}$$

**Table 3:** Chemical composition of concentrate and hay and diet fed

Item	Step 1	Step 2
Ingredient (g/d as fed)		
Hay	1800	1500
Concentrate	50*	300
Pasture	-	+
Chemical composition of hay		
DM (g/kg, as fed)	860	
CP (g/Kg DM)	180	
CF (g/Kg DM)	350	
Fat (g/Kg DM)	20.0	
Ash (g/Kg DM)	9.00	
DE (Mcal/kg DM)	2.120	
Chemical composition of concentrate		
DM (g/kg, as fed)	880	
CP (g/Kg DM)	160	
CF (g/Kg DM)	110	
Fat (g/Kg DM)	45.0	
Ash (g/Kg DM)	89.6	
Vitamin A (UI/Kg DM)	2227	
Vitamin D3 (UI/Kg DM)	454	
Choline (mg/Kg DM)	196	
Pantothenic acid (mg/Kg DM)	11.4	
Vitamn E (mg/Kg DM)	45.4	
Vitamin B1 (mg/Kg DM)	14.2	
Vitamin K (mg/Kg DM)	2.84	
Vitamin B6 (mg/Kg DM)	3.40	
Vitamin B12 (mg/Kg DM)	0.017	
DE (Mcal/kg DM)	3.907	

Energy density for concentrate and forage was predicted by the formula of NRC (NRC 2007) for horses.

Four weeks after the beginning of the second phase, additional blood tests were conducted. The parameters of the CBC and biochemical analysis improved.

The patient’s BCS increased from 3 to 4/9, and her weight reached 112 kg. The estimated daily energy (DE, Mcal) and protein (g) intake considered the voluntary feeding behaviour of the jenny. In this second phase, hay consumption per day reached and, in rare cases, exceeded 1.2 kg, apparently contributing to cover requirements for 2.19Mcal of DE and 186 g of protein supply, along with an average daily compound feed consumption of 300 g, contributing for 1.03 Mcal and 42.2 g of protein supply. The average daily gain (ADG) pointed to 0.53 kg BW/d throughout the second phase. Indeed, the jenny voluntary fed on straw and had free access to pasture (which increased in time as the lame condition was resolved). It is however typical of donkeys at our latitude feeding preferably on hay rather than grazing on fresh grass as available feeding sources, which contributed to residually cover energy and protein daily requirements. However, it is to underline that the equation applied for the estimation of digestible energy density in terms of Mcal/kg DM of hay and compound mixed concentrate feeds is referred to horses. Independently on estimation of requirements based on daily DM intake capacity as percentage of BW (max 2% at maintenance) and energy requirements per kg of metabolic weight of the donkey, the efficiency in digesting and utilizing energy of feedstuffs differs between horses and donkeys, being the latter generally more efficient in degrading fibre (including lignified fractions, thanks to favourable microbial composition of the large intestine, richer in *archaea*, in association with longer transit time) and likely equipped with a more assimilative intestine (Cuddeford et al. 1995; Wood et al. 2005).That way, the estimation of DE density of feedstuff for horses with current equation may lead to overfeeding.

The wounds appeared completely healed, following a second purpose scarring process; the coat quality also improved, appearing uniform, shiny and thicker, with new hairs showing appropriate colour for the breed standard. The faecal score also turned to improve markedly (firm and well-shaped, light brown-coloured scybala).

### Follow-up

**Fig. 1:**
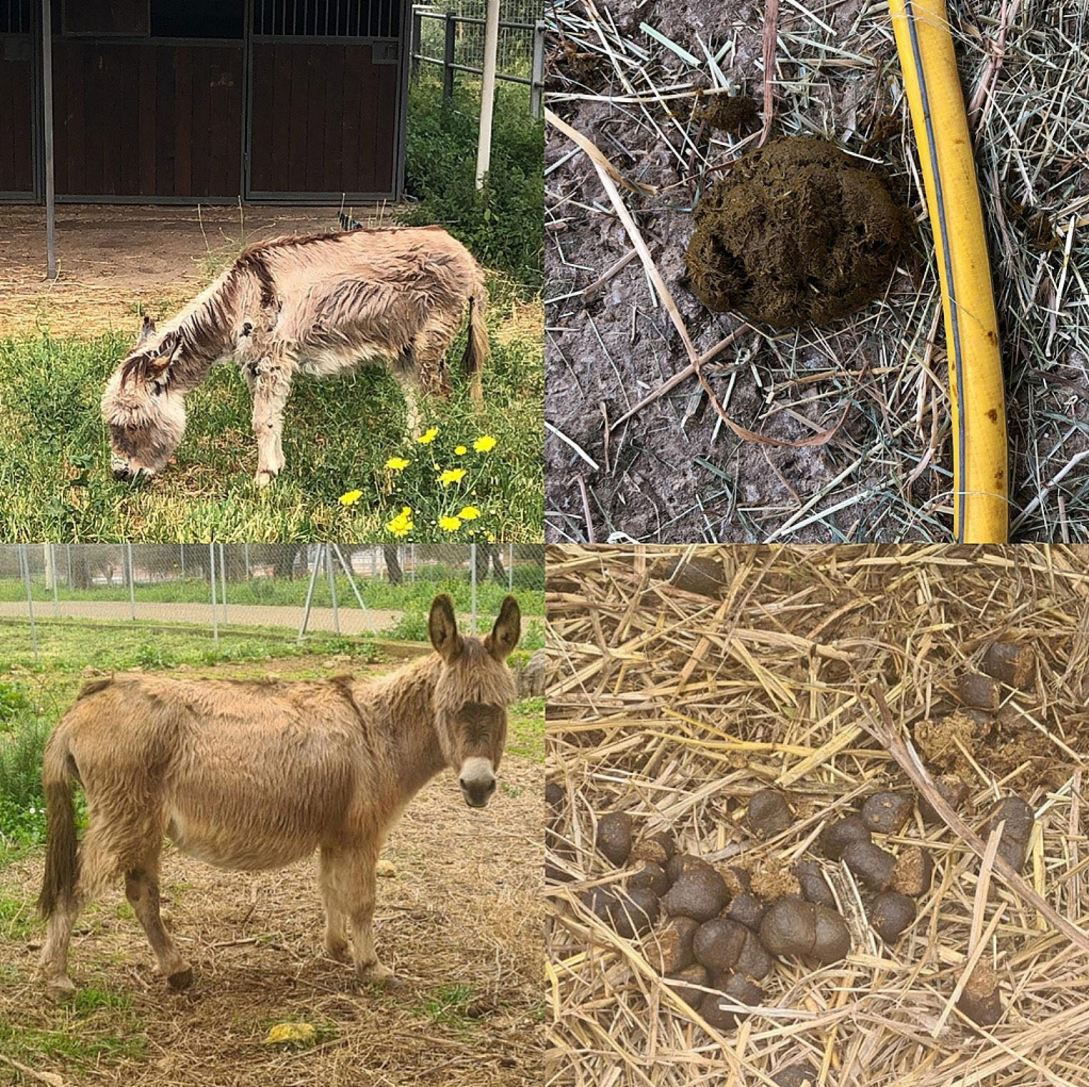
The image displays the condition of the jenny during the therapy and at follow up. Top left: D1, the donkey weighed 88 kg. Top right: The initial faecal score is 2/8, suggesting loose faeces. Bottom left: Follow up, the jenny’s weight increased to 118 kg. Bottom right: The concluding faecal score improved to 5/8

The recovery of the jenny through this nutritional approach associated with medications proved effective and allowed the reintroduction into the group of donkeys permanently hosted in the paddock of the teaching farm of the VTH, to care for welfare through intraspecific interaction(De Santis et al. 2021).Overall, general physical conditions improved (Fig. 1), intestinal motility was restored. Mucous membranes of natural openings turned normal with appropriate capillary refill time, and lameness reduced from a score of 4 to 1. Wounds progressed to complete healing by second intention. Stable BCS, optimal FS and overall clinical conditions were assessed after the patient was moved to the group of donkeys. After 30 weeks, the follow-up required to monitor the BCS, MCS and FS, with CBC and biochemical profile, that turned out to drop within the reference values (D3, Tables 1 and 2). The animal weighed 118 kg and showed a BCS of 5/9, MCS of 3/5 and a FS of 5/8.

## Discussion and conclusions

Feeding management is crucial for the recovery of the emaciated donkey. When feed intake is limited, and energy intake does not meet requirements, donkeys are prone to experience a life-threatening condition called hyperlipaemia, a disorder that mostly affects ponies, donkeys, and miniature horses (Dunkel and McKenzie 2003). It is characterized by a rise in serum triglyceride values more than 5.65 mmol/l, as well as lipaemia and fatty infiltration of the liver or other organs (Burden et al. 2013). There are various factors that increase the risk of hyperlipaemia in donkeys and other equids. These include obesity, late pregnancy (Cappai et al. 2021), lactation, lack of exercise (Durham et al. 2015), and advancing age. Stress is another crucial factor that can lead to a reduction in appetite, even with minor stressors, in susceptible individuals. Furthermore, underlying disease states such as colic, choke, dental disease, respiratory tract infection, and conditions associated with significant pain can also trigger a decrease in appetite (Harrison and Rickards 2018). The nutritional history of this jenny is missing, since the jenny used to graze freely in the park with a large group of more than fifty donkeys, and likely got lame and lost weight progressively. Lame animals are known to reduce the feed intake, due to reluctance to move. The reduced appetite observed in this extremely emaciated jenny was likely conditioned by the hyperlipaemia. The onset of the metabolic syndrome follows a low energy intake from the diet. In such condition of negative energy balance (low feed intake in contrast to energy requirements), the body fat depots are mobilized, and free fatty acids are released in the bloodstream to be used as endogenous energy source. The systemic high demand for energy can overload the hepatocyte, which may start to store excess of fats in the intracellular environment. The failure in processing fats for energy purposes can lead to the conversion into ketone bodies (playing as anorexigenic molecules) and to jaundice (observed in the donkey at arrival). The nutritional objectives were therefore oriented to different purposes: on one side, the feeding treatment aimed to re-establish the voluntary feed intake in sufficient amounts to progressively cover energy requirement; on the other side, the re-feeding syndrome should be avoided (Bookbinder and Schott 2nd 2021). Usually, the best course of action is to restore the patient appetite with feed that it finds more appealing. In addition, the research indicates that hand feeding may also be effective, since donkeys frequently eat from the hand of a trusted owner more easily than from a bucket (Burden et al. 2013). In this case, however, the patient came from a park where donkeys are not used to be handled by humans. Although she was not used to people, it was decided to provide the diet through a feeder and limit (at least in the first phase) human interactions to time required for medications. Several clinical reports from the literature describe the use of parenteral feeding to treat hyperlipaemia in donkeys (Durham 2006; McKenzie 2015). In our case, the patient exhibited a prompt response to the nutritional intervention, demonstrating voluntary feeding behaviour, albeit with limited feed intake at start. Consequently, a primarily enteral dietary intervention was chosen, with a supplement containing appropriate vitamins with fluid administration. Where enteral feeding is possible, this offers to limit the complication from other secondary problems, chiefly related to luminal microbial activities of the large intestine. Indeed, prolonged period of starvation is associated to the loss of intestinal barrier integrity with a higher chance to the onset of the “leaky gut” syndrome (Stewart et al. 2017). Idiopathic typhlocolitis could not be excluded for no full abdominal scan was conducted. However, the anamnestic data on the group of donkeys living with the jenny in the sanctuary, pointed to her as an isolated case, likely due to the extreme emaciation following the lameness, from the external lesion of the arm (Du Toit et al. 2010). Additionally, scientific evidence about the role of fibre rich diets in keeping the intestinal mucosal barrier is also provided, in spite of starch rich diets (Raspa et al. 2021), in horses. Still evidence in donkeys is to be proved. In conclusion, the two-step nutritional approach had remarkable results, with complete clinical and nutritional recovery of the patient, beginning from a severe condition of compromised health status.

## Data Availability

No datasets were generated or analysed during the current study.
